# Precision of photon-counting detector CT compared to conventional CT for total wrist arthroplasty migration: a cadaveric and clinical study

**DOI:** 10.1007/s00256-026-05293-1

**Published:** 2026-07-28

**Authors:** Gustav Lind, Erik Tesselaar, Olof Sandberg, Simon Farnebo

**Affiliations:** 1 Department of Biomedical and Clinical Sciences, Faculty of Medicine and Health Sciences, Linköping University, Linköping, Sweden; 2 Department of Plastic Surgery, Hand Surgery, and Burns, Linköping University, Linköping, Sweden; 3 Department of Medical Radiation Physics, Department of Medical and Health Sciences, Linköping University, Linköping, Sweden; 4 Center for Medical Image Science and Visualization, Linköping University, Linköping, Sweden; 5 Sectra, Linköping, Sweden

**Keywords:** Computed tomography, Photon-counting, Total wrist arthroplasty, Micromotion, CT-RSA

## Abstract

**Objectives:**

Implant loosening is a serious complication after total wrist arthroplasty. Radiostereometric analysis has been the gold standard for measurement of implant migration; however, the use of bead free computer tomography radiostereometric analysis (CT-RSA) has emerged as an alternative due to its higher precision. In this study, the precision of a novel photon-counting detector CT (PCD-CT) was compared to conventional energy-integrating detector CT (EID-CT) at different radiation doses.

**Methods:**

One cadaveric wrist with total wrist arthroplasty (TWA) implant was scanned 40 times with EID-CT and PCD-CT at two radiation doses. Additionally, eight patients with the same implant were scanned with PCD-CT post-surgery, and after 6 and 12 months. PCD-CT scans were reconstructed with 0.2-mm and 1.0-mm slice thickness. Precision data for the cadaveric wrist and migration data for the patients were calculated using CT-RSA.

**Results:**

The precision of total implant rotation was significantly better with PCD-CT at half the radiation dose (0.08°) compared to full-dose EID-CT (0.15°, *p* = 0.002). Similarly, translational precision at the implant tip was higher with half-dose PCD-CT (0.02 mm) than with EID-CT (0.03 mm, *p* = 0.04). For migration, the proximal component had moved (median) −0.04 mm distally at 6 months and 0.01 mm at 12 months. The rotation around the axis at these time points was 0.03° and 0.06°, respectively.

**Conclusions:**

In this cadaveric precision study, PCD-CT enabled CT-RSA measurements with high precision at a lower radiation dose than conventional EID-CT. The accompanying eight-patient in vivo cohort should be interpreted as a feasibility cohort and showed only small descriptive early migration values during the first postoperative year. Larger longitudinal studies are needed to determine clinically relevant migration thresholds and the value of this method for detecting implant loosening.”

**Relevance statement:**

Photon‑counting detector computed tomography and computed tomography micromotion analysis allow high-definition tracking of total wrist arthroplasty migration at half the radiation dose of conventional CT, offering a high‑precision, low‑dose tool for early detection of implant loosening.

## Introduction

Total wrist arthroplasty (TWA) has emerged as an effective surgical movement-preserving alternative to wrist fusion for patients with severe wrist osteoarthritis [1, 2]. Historically, wrist implant survival and complication rates have been unsatisfactory, but recent studies show a 10-year survival in between 80 and 90% of the recent fourth generation prosthesis [2]. Still, approximately 30% need revision and/or correction surgeries, in many cases due to micromovement and migration of the prosthesis fixation [1, 3]. Observation of early signs of migration has been shown to be important for early detection of such changes in implant surgeries for the hip and knee [4, 5]. Today, the gold standard for detecting micromotion in orthopedic implants is radiostereometric analysis (RSA), offering highly precise migration data by using tantalum markers implanted in the bone as position reference [6]. A relatively new CT-based three-dimensional technique, computed tomography radiostereometric analysis (CT-RSA), detects movements by using unique anatomic structures in the bone as position reference when comparing sequential CT scans. This noninvasive technique has shown precision comparable to conventional RSA in selected orthopedic applications, although data for wrist arthroplasty remain limited [6, 7]. Only a few studies have been published analyzing this technique for wrist implant migration.

Recently, photon-counting detector CT (PCD-CT) has become clinically available. The PCD-CT technique, due to its detector design, improves spatial resolution and dose efficiency and gives lower image noise compared to energy-integrating detector CT (EID-CT) [8, 9]. Recent studies have shown that the PCD-CT technique yields improved diagnostic quality in many different medical areas [10, 11]. There is insufficient information in the literature, however, about the value of PCD-CT in imaging of wrist joint implants and no study using it in combination with CT-RSA.

In this study, we assessed if PCD-CT compared to EID-CT has a higher precision in measuring implant migration with CT-RSA. This was done by sequentially examining one cadaver arm. We also investigated whether radiation doses can be reduced when PCD-CT is used without reducing precision. An eight-patient feasibility cohort was also recruited to assess migration of TWA at one year, using both modalities.

## Method

### Study design and participants

In this prospective study, one cadaveric wrist and eight patients underwent total wrist arthroplasty (TWA) with the Motec TWA (Swemac Innovation AB, Linkoping, Sweden). The cadaveric specimen consisted of an intact upper extremity with bones and soft tissues fully retained from the fingers to the elbow, disarticulated at the cubital joint. No external fixation was used during scanning, and the specimen was repositioned in a random fashion between acquisitions to simulate repeated patient examinations and enable precision estimation. Information on age and sex of the cadaveric specimen was not available.

Eight consecutive patients that were on the waitlist for primary Motec total wrist arthroplasty were invited to enroll in the study (Table 1). All patients were found eligible as they were able to undergo postoperative and follow-up CT examinations according to the protocol. No patient dropped out of the study. Exclusion criteria were revision arthroplasty, inability to complete follow-up CT examinations, severe imaging artefacts precluding CT-RSA analysis, or withdrawal of consent. The cadaveric specimen was included if the Motec implant could be inserted and repeated CT examinations could be performed without gross mechanical instability. Baseline demographic and clinical characteristics of the in vivo cohort, including follow-up completeness, are summarized in Table 1.

**Table 1:** Baseline demographics of the included in vivo patients. All cases were included consecutively and all completed CT scans at 2 days postop (first scan, mean), 185 days (second scan, mean), and 367 days (third scan, mean). SLAC = scapho-lunate advanced collapse, DRF = distal radius fracture

Case nr	Age at surgery	Diagnosis	Gender (male/female)	Laterality (left/right)	Time to first postop CT (day)	Time to second postop CT (day)	Time to third postop CT (day)
1	63	SLAC	M	L	1	183	366
2	54	SLAC	M	R	1	181	359
3	63	DRF	F	L	2	175	371
4	66	SLAC	F	R	6	181	364
5	72	SLAC	F	R	0	203	337
6	69	SLAC	M	R	5	181	365
7	59	SLAC	M	R	1	190	413
8	75	SLAC	F	L	1	188	358

The surgeries were performed by the same experienced surgeon. All patients were scanned postoperatively (minimum 1 day, maximum 5 days) and at 6 and 12 months after surgery. The study was approved by the Swedish Ethical Review Authority (Dnr 2023–04144-01).

### Implant description

**Fig. 1:**
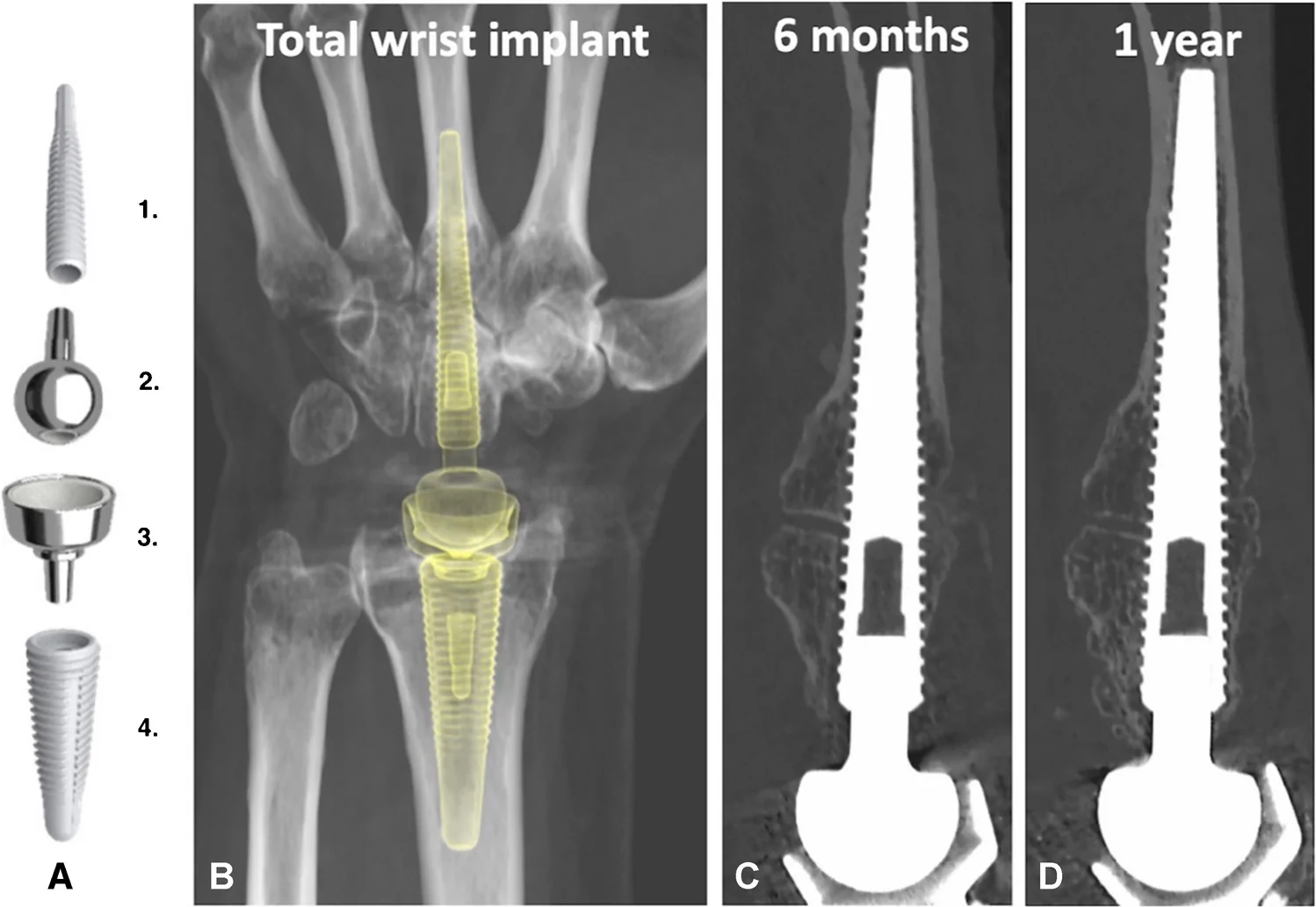
**A** The Motec Wrist arthroplasty with four separate modular parts: (1) distal screw, (2) ball, (3) polyethylene-coated socket, and (4) proximal screw, that can be changed to finesse the tension of the final implant position. **B** 3D reconstruction of a PCD-CT scan 1 year after implantation. Clinical examples of PCD-CT images of a fixed implant after 6 months (**C**) and 12 months (**D**), respectively

The Motec prosthesis is a modular, calcium-phosphate-coated titanium implant composed of four parts including a ball, a polyethylene-coated socket, and two screws. Each component moves independently, although the distal screw is fixed through the capitate into the third metacarpal, and the proximal screw is fixed in the radius. Successful osseointegration of the screws in bone and free movement of the ball in the socket, without excessive tension, are critical for optimal function [1]. A schematic of the implant and its components is shown in Fig. 1.

### Imaging protocol

State-of-the-art EID-CT (SOMATOM Force, Siemens Healthineers, Forchheim, Germany) and PCD-CT (NAEOTOM Alpha, Siemens Healthineers, Forchheim, Germany) scans were performed of both the cadaveric wrist and the patients.

The cadaveric wrist was scanned 40 times with EID-CT at a fixed CTDIvol of 7.5 mGy and 40 times with PCD-CT at both matched and half-dose levels. Manual repositioning of the cadaveric specimen was performed between each scan, and from these images, 20 double-exam sets were created for both EID-CT and PCD-CT. The two scanners reference CTDIvol to different standard phantoms: the 16-cm phantom for the SOMATOM Force (CTDIvol,16) and the 32-cm phantom for the NAEOTOM Alpha (CTDIvol,32). For the same radiation output, CTDIvol,16 ≈ 2 × CTDIvol,32 (AAPM Report 204); the console values are therefore not directly comparable between scanners. To report a patient-size-adjusted dose that is comparable across scanners, the size-specific dose estimate (SSDE) was calculated as CTDIvol,32 × fsize, where fsize = 2.76 for an effective wrist diameter of approximately 8 cm (AAPM Report 204, Table 1D). This yields SSDE = 10.35 mGy for the full-dose protocols on both scanners and 5.18 mGy for the half-dose PCD-CT arm.

For the patients, EID-CT was acquired using a clinical protocol with automated tube current modulation (CARE Dose4D) (Table 2) (CTDIvol,16: 7.2 ± 0.1 mGy, SSDE: 9.9 ± 0.1 mGy). For PCD-CT, the radiation dose was matched to the EID-CT scans (CTDIvol,32: 3.7 ± 0.1 mGy, SSDE: 10.2 ± 0.3 mGy). Both systems used an ultra-high-resolution mode.

**Table 2:** A detailed overview of the acquisition and reconstruction parameters used for the radiologic exams

CT technology*	EID-CT	PCD-CT
Scanner platform	SOMATOM Force	NAEOTOM Alpha
Tube voltage (kVp)	Sn150	Sn140
CTDIvol (mGy)**	7.5 (cadaver) 7.2 ± 0.1 (patients)	3.8 (cadaver, full dose) 1.9 (cadaver, half dose) 3.7 ± 0.1 (patients)
SSDE (mGy)***	10.4 (cadaver) 9.9 ± 0.1 (patients)	10.4 (cadaver, full dose) 5.2 (cadaver, half dose) 10.2 ± 0.3 (patients)
Dose modulation	CARE Dose4D	None, matched to EID-CT
Collimation (mm)	2 × 64 × 0.6 mm (38.4 mm)	120 × 0.2 mm (24.0 mm)
Spiral pitch	0.6	0.4
Rotation time (sec)	1.0	1.0
Reconstruction kernel	Ur77	Br89
Iterative technique/strength	ADMIRE 3	QIR 3
Slice thickness/increment (mm)	0.4/0.2	0.2/0.1
Field of view (mm)****	100–120	100–120
Reconstruction matrix size	512 × 512	1024 × 1024

*Siemens Healthineers, energy-integrating detector (EID) CT: Syngo CT VB10; photon counting detector (PCD) CT: Syngo VA50A

**CTDIvol as displayed on the scanner console: referenced to the 16-cm PMMA phantom for EID-CT (SOMATOM Force) and to the 32-cm PMMA phantom for PCD-CT (NAEOTOM Alpha). These values are not directly comparable between scanners; see SSDE below

***SSDE: size-specific dose estimate, calculated as CTDIvol,32 × fsize (AAPM Report 204, Table 1D). For the force, CTDIvol,32 = CTDIvol,16/2. fsize = 2.76 for an effective wrist diameter of ~ 8 cm. SSDE is independent of the reference phantom and directly comparable between scanners

****Same FoV for both scanners; as narrow as possible

During CT acquisition, the wrist was positioned in a standardized neutral position. The hand and forearm were supported and immobilized using positioning aids to minimize motion during scanning.

EID-CT scans were reconstructed at a slice thickness of 0.4 mm using the Ur77 reconstruction kernel optimized for bone imaging. PCD-CT scans were reconstructed at slice thicknesses of 0.2 mm and 1.0 mm using the Br89 reconstruction kernel optimized for bone imaging. The 1.0-mm slice thickness was included because this represents a commonly used clinical reconstruction setting for PCD-CT, whereas 0.2 mm represents an ultra-high-resolution research reconstruction. Including both allowed us to assess whether the increased precision observed with PCD-CT is preserved under clinically realistic conditions*.* A detailed overview of the acquisition and reconstruction parameters is shown in Table 2.

### CT-RSA analysis

**Fig. 2:**
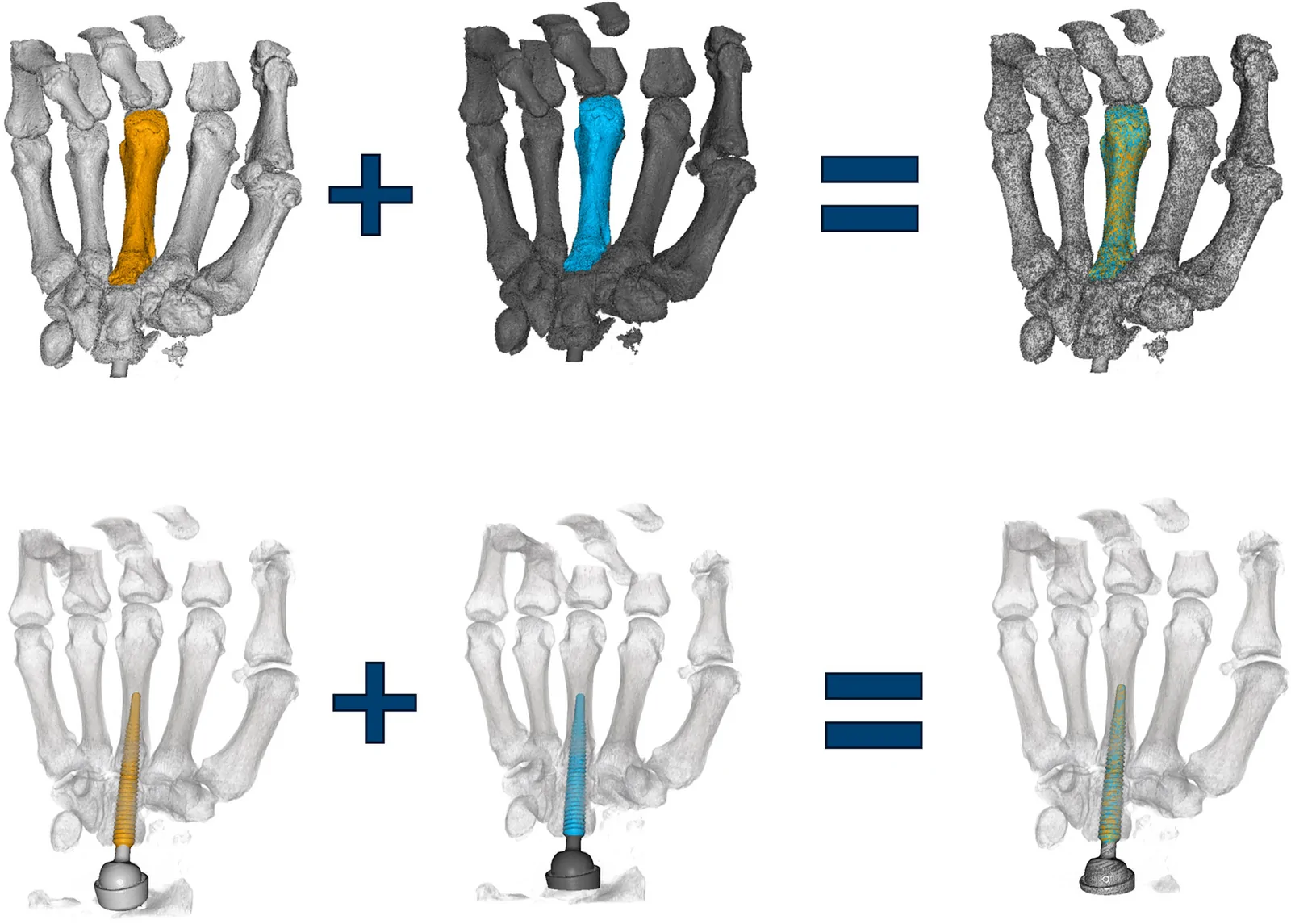
The principle of the CT-RSA software algorithm to find the migration of an implant relative to bone over time. First, the bone (metacarpal III) is indicated in post-op CT (white) and follow-up CT (dark gray) and merged (zebra striped). The same is done for the implant

Precision and migration data were calculated using CT-RSA software (CTMA, Sectra, Sweden). The process is summarized in Fig. 2. For step 1, the stationary body (third metacarpal) is identified in both CT scans and merged. For step 2, the implant is indicated and merged in both CTs. For step 3, coordinate system definition and measurement points are defined. Thereafter the change in position of the implant relative to the bone is quantified automatically. For the precision estimates of the cadaveric wrist, the distal component was analyzed.

Precision was quantified as 1.96 × standard deviation (SD) from repeated measurements, corresponding to the 95% limits of agreement, which is a commonly used definition of precision in micromotion and RSA studies. Segmentation and registration were performed semiautomatically, with a visual observer indicating the anatomical regions and setting a threshold value. The resulting automated registrations were then reviewed by an experienced observer to ensure technical plausibility. The observers were not formally blinded to scan protocol or time point, and no repeated analyses were performed to quantify intra- or interobserver variability.

**Fig. 3:**
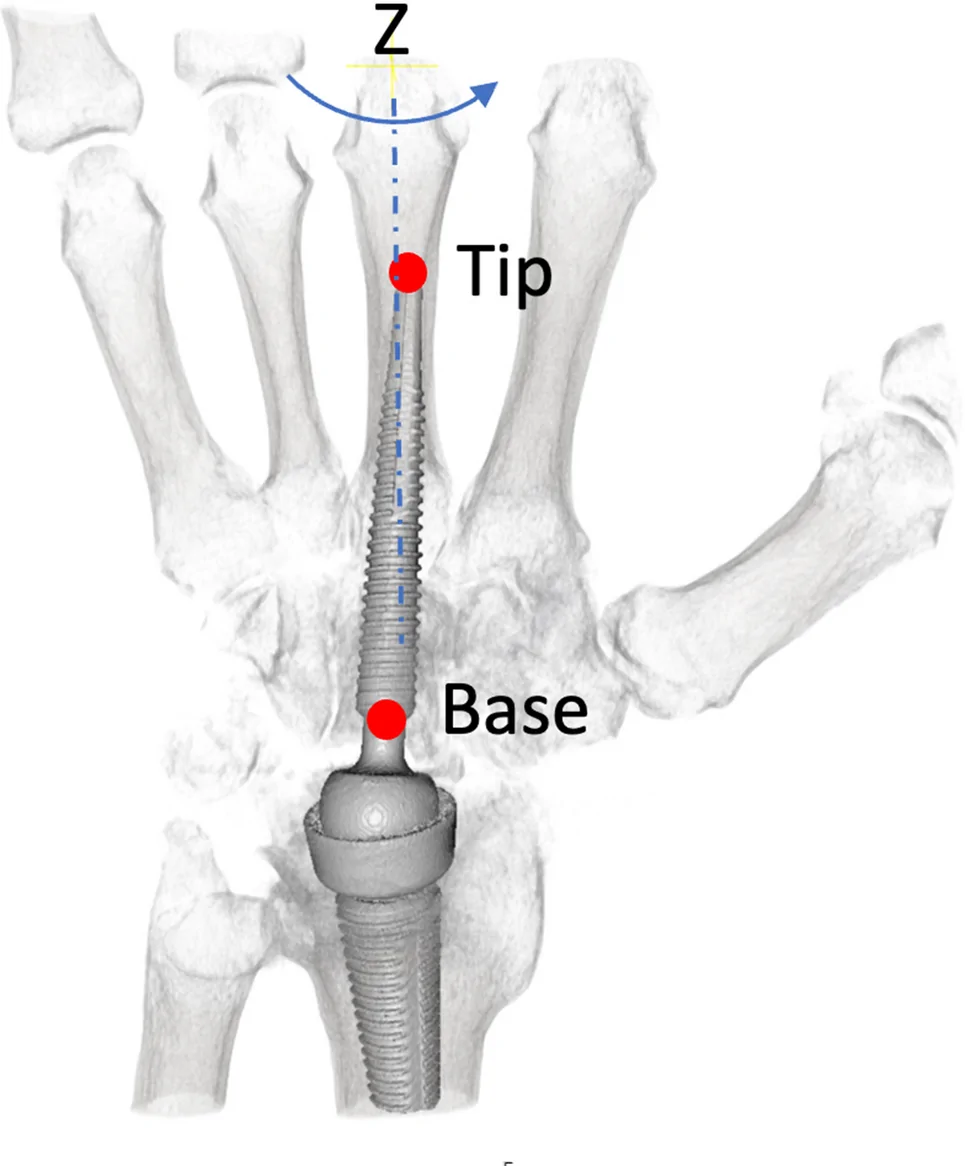
Migration was assessed for both the tip (T) and base (B) of the prosthesis and was expressed in terms of rotation (Z) and translation, relative to the bone

Migration was assessed for both the tip and base of the prosthesis and was expressed in terms of rotation and translation, see Fig. 3, relative to the bone. Positive values indicate distal migration, whereas negative values indicate proximal migration. In this study, translation was defined along three orthogonal axes of a right-handed coordinate system embedded in the CT volume, with the proximal–distal axis aligned to the implant screw axis. From this, migration is reported as vector components.

### Statistical analysis

All statistical analyses were performed using GraphPad Prism (version 10.4.0 for macOS, GraphPad Software, Boston, MA, USA).

For the cadaveric study, one-way analysis of variance (ANOVA) was done using the individual repeated-scan measurements of translation and rotation to compare precision between imaging protocols. Šídák’s post hoc tests were used to correct for multiple comparisons. Assumptions of normality (Shapiro–Wilk test) was evaluated prior to conducting ANOVA.

For the patient study, migration data are reported as medians and interquartile ranges (IQRs), and 95% confidence intervals were computed for precision estimates. A *p* value < 0.05 was considered statistically significant.

## Results

For the cadaveric wrist, photon-counting detector CT (PCD-CT) demonstrated superior precision compared with energy-integrating detector CT (EID-CT).

The precision (upper 95% CI) of total implant rotation was 0.08° with half-dose PCD-CT, compared with 0.15° for full-dose EID-CT (*p* = 0.002). Similarly, the precision of translation at the implant tip was 0.02 mm with half-dose PCD-CT, compared with 0.03 mm for EID-CT (*p* = 0.04).

Across all protocols, rotational precision ranged from 0.02° to 0.10° for PCD-CT and from 0.04° to 0.32° for EID-CT, while translational precision ranged from 0.02 to 0.07 mm and 0.02 to 0.03 mm, respectively. Detailed precision values for all dose and slice-thickness combinations are summarized in Table 3.

**Table 3:** CTMA precision, mean and 1.96*SD in mm of observed translation and rotation in 20 × 2 repeated EID-CT and PCD-CT scans of a cadaveric wrist distal component using different radiation doses and slice thicknesses. t-t = total translation, t-rot = total rotation, z-rot = rotation around the proximal/distal axis

	t-t base (mm)		t-t tip (mm)		t-rot (°)		z-rot (°)	
	Average	1.96×SD	Average	1.96×SD	Average	1.96×SD	Average	1.96×SD
EID-CT full dose 0.6 mm	0.04	0.02	0.03	0.02	0.15	0.19	−0.05	0.32
PCD-CT full dose 0.2 mm	0.05	0.06	0.02	0.02	0.08	0.08	−0.01	0.10
PCD-CT full dose 1.0 mm	0.05	0.06	0.03	0.03	0.12^§^	0.12	0.02	0.22
PCD-CT half dose 0.2 mm	0.05	0.07	0.02*	0.02	0.08*	0.09	0.03*	0.10
PCD-CT half dose 1.0 mm	0.05	0.06	0.03	0.03	0.12^§^	0.11	0.03	0.20

*Sign. difference compared to EID-CT

^§^Sign. difference compared to 0.2 mm

**Fig. 4:**
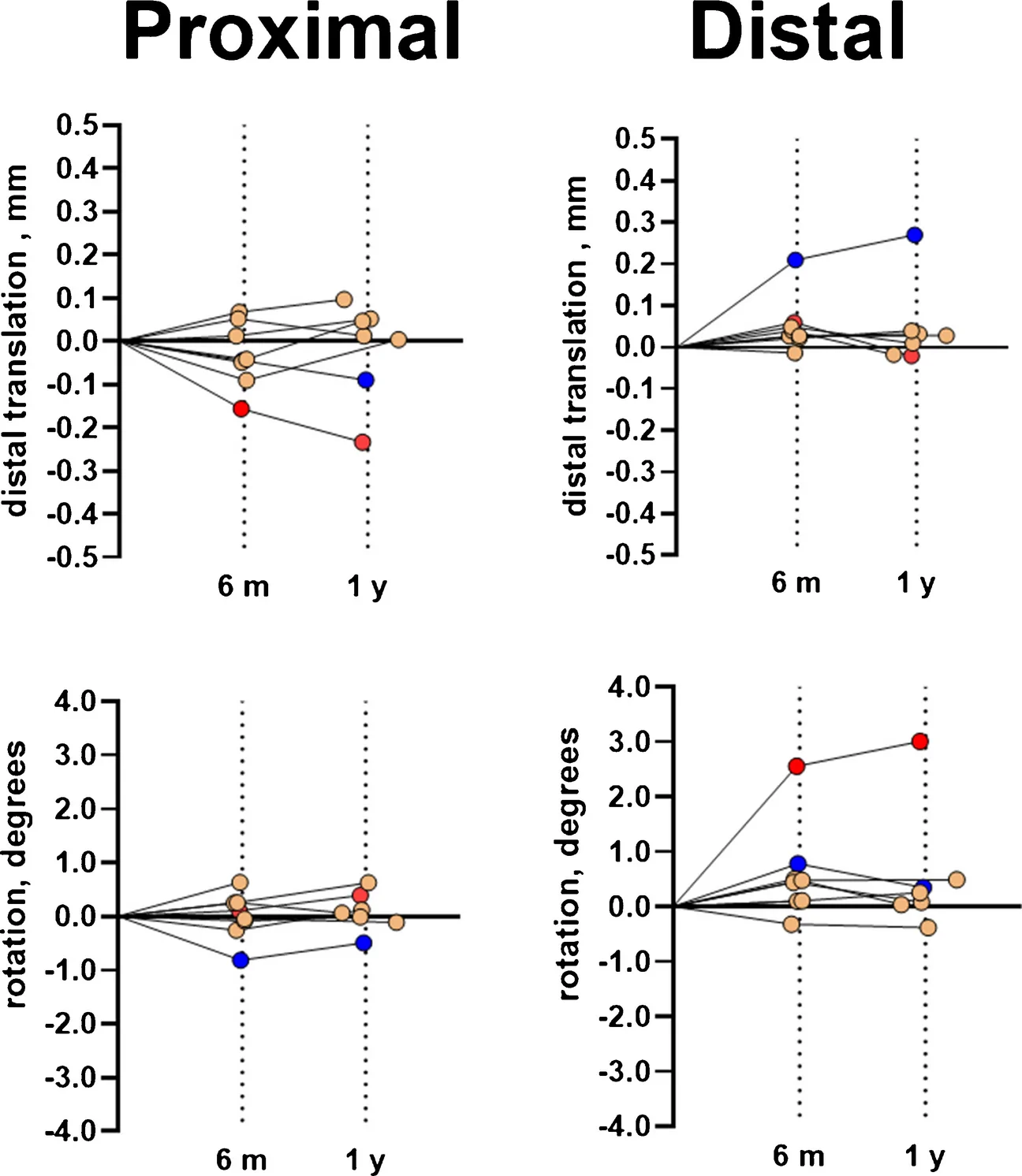
The proximal/distal translation and rotation for the proximal and distal component is shown from post-op to 1 year. Blue and red colors are used to track two specific patients across the four graphs. Corresponding 95% precision limits in the cadaver were 0.1° and 0.02 mm

For the patients (all data from PCD-CT), as measured by median (LQ, UQ), at 6 months the base of the proximal component had moved −0.04 mm (−0.08, 0.05). At 12 months, it had moved 0.01 mm (−0.09, 0.06). The total translation at these two time points was 0.10 mm (0.06, 0.16) and 0.10 mm (0.07, 0.22), respectively. The rotation around the proximal/distal axis at these two time points was 0.03° (−0.21, 0.25) and 0.06° (−0.11, 0.45). The total rotation was 0.29° (0.14, 0.35) and 0.25° (0.13, 0.48). For the distal component, the base had moved 0.04 mm (0.02, 0.06) and 0.02 mm (−0.02, 0.04) at 6 and 12 months. The corresponding total translation was 0.09 mm (0.07, 0.21) and 0.15 mm (0.07, 0.23). The rotation around the proximal/distal axis was 0.44° (0.10, 0.71) and 0.17° (−0.07, 0.49) at 6 and 12 months. The total rotation was 0.45° (0.31, 0.82) and 0.35° (0.27, 0.53). These results are illustrated in Fig. 4.

## Discussion

The results of this study indicate that the CT-RSA technique used in a cadaveric model of a TWA has a high precision in detecting migration both using EID and PCD-CT. In addition, PCD-CT demonstrated superior precision in measuring migration at a lower radiation dose.

Previous experimental and comparative studies have been evaluating the accuracy and validity of CT-RSA, mainly in the lower extremities [7, 12, 13]. Brodén et al. showed measurements for accuracy for the CT-RSA in acetabular cups ranging between 0.07 and 0.32 mm for translations and 0.21 and 0.82° for rotation [14]. In a later study, they compared the precision of CT-RSA directly with conventional radiostereometric analysis (RSA) in cemented acetabular cups and reported precision between 0.10 and 0.16 mm for translation and 0.31°–0.37° for rotation. The precision of RSA was 0.09–0.26 mm for translation and 0.43–1.69° for rotation, demonstrating comparable precision [7]. Similar precision levels have been reported in experimental studies also in the upper extremities such as Brodén et al. for shoulder arthroplasty models [15], and Lundqvist et al. for distal radius fractures treated with locking plates [16], supporting the validity of CT-RSA for implant migration assessment [15]. In hand and wrist applications, Wilcke et al. demonstrated CT-RSA data on movement between the scaphoid and lunate during the dart-throwing movement [17] and how triquetral movement is impaired after lunocapitate arthrodesis [18]. There are very few studies published on wrist implant migration using CT-RSA, including one pilot study by Sagerfors et al. on three TWA patients, where stress CTs were acquired [19]. Their results indicated a possible role of CT-RSA as diagnostic support for suspected aseptic loosening. Further, Holm and co-workers measured precision and accuracy with RSA in 10 phantoms and 30 patients with TWA who were examined twice. The translation precision in the phantom study ranged from 0.03 to 0.14 mm and for rotation from 0.18 to 1.52°, with slightly higher values in the clinical setting, ranging from 0.06 to 0.18 mm for translation and from 0.32 to 2.18° for rotation [6].

By comparison, our study showed favorable precision values for translation with CT-RSA of 0.05 mm for both EID-CT and PCD-CT with half dose and 1.0-mm thickness slices. For rotation, the precision was 0.12° for PCD-CT and 0.15° for EID-CT.

Importantly, the aim of this study was methodological, focusing on measurement precision and feasibility rather than defining clinically relevant migration thresholds. The small absolute migration values observed in vivo should therefore be interpreted in relation to the measured precision limits, rather than as indicators of clinical loosening. The in vivo cohort of eight patients was included primarily to demonstrate clinical feasibility and to characterize early implant behavior during the first postoperative year, rather than to provide statistical validation of precision. In these patients, the Motec arthroplasty components show very small movements during the first year after implantation, aligning with findings from earlier studies using RSA [6, 7]. This indicates that there is minimal prosthesis movement during the first year.

The primary difference between PCD-CT and state-of-the-art EID-CT lies in their detector technology. EID-CT employs energy-integrating detectors (EIDs), which convert incoming X-ray photons into electrical signals through an indirect two-step process. First, photons are absorbed by a scintillator layer and converted into visible light pulses. These light pulses are then detected and transformed into electrical signals by a photodiode array. To prevent light scattering between adjacent detector elements, septa are placed between them, which limits the minimum possible size of individual detector elements.

In contrast, PCD-CT utilizes photon-counting detectors, where X-ray photons are directly converted into electrical signals within a semiconductor layer. This direct conversion eliminates the need for a scintillator and septa, allowing for smaller detector elements, higher spatial resolution, and improved dose efficiency compared to EID-CT [9, 10]. The benefits of PCD-CT over EID-CT have previously been shown in cadaveric wrists, showing that PCD-CT improves image quality in terms of higher spatial resolution, even at 50% radiation dose [20]. This suggest that PCD-CT may enable more exact diagnosis of subtle fractures and other better visualization of bone with decreased mineral density or osteopenia and more accurate assessment of fracture healing [8]. In another study, Kämmerling et al. compared visualization of the healing process of scaphoid fractures, which showed superior visibility of bone microstructure with PCD-CT compared with EID-CT [11]. The use of photon-counting detector CT for hand and wrist surgery has previously been reviewed [21].

In this study, PCD-CT with full dose and with 0.2-mm thickness slices had the best general precision. The higher precision rates, and the fact that you do not need invasive implanting of metal beads, further emphasize that CT-RSA has an advantage for measuring wrist implant migration compared to RSA. For the 1-year patient follow-up, we had minimal micromotion in the first 6 months, except for translation in one patient (0.3 mm) and for rotation in another patient (3°).

PCD-CT with full dose and the thinnest 0.2-mm thickness slice had significant superior precision measuring migration over EID-CT. This is true also for half dose with 0.2-mm slices. With half dose and thicker 1.0-mm thickness slices, we had comparable precision as the EID. Even though the risk is small using CT scan for the wrist, it remains essential to minimize radiation exposure to reduce the risk of harmful effects according to the ALARA (as low as reasonably achievable).

This study demonstrates the quality and precision of the CT-RSA technique regarding wrist joint Motec prosthesis. A limitation in the study is the small number of patients included and the limited follow-up period of 1 year. This makes it difficult to generalize the results regarding the level of migration for this patient group as loosening may occur over time. Because we had no intention to draw conclusions regarding patient function related to micromovements of the implants in this study, we did not include patient outcome measures. The primary purpose of including the patient cohort was rather to demonstrate the feasibility of using PCD-CT with CT-RSA for assessing wrist implant migration with high precision in vivo. While our findings confirm that the method can reliably detect very small micromotions, we acknowledge that a minimum follow-up of at least 5 years together with clinical outcome measures is required to adequately evaluate implant stability, survival, and long-term clinical value of implant loosening.

Although the repeated cadaveric scans provided robust estimates of technical precision, we did not perform formal intra- or interobserver variability analyses. This is a limitation because the CT-RSA workflow includes semiautomatic steps, including anatomy selection and thresholding, that may be influenced by observer input. Therefore, the present study primarily characterizes scan-rescan precision rather than the full reproducibility of the observer-dependent workflow. Future studies should include repeated analyses by the same and independent observers to quantify intra- and interobserver variability. Also, because we only used one cadaveric arm, the 20 double-exam sets were treated as repeated technical measurements of the same specimen and not as independent biological samples. Therefore, the statistical comparisons should be interpreted as comparisons of technical measurement variability between protocols rather than necessarily being generalized to a wider population.

Lastly, as the study focused on a specific implant type, the findings cannot be directly extrapolated to other wrist arthroplasties or joint arthroplasties in general.

## Conclusion

In this cadaveric precision study, PCD-CT enabled CT-RSA measurements with high precision at a lower radiation dose than conventional EID-CT. The accompanying eight-patient in vivo cohort should be interpreted as a feasibility cohort and showed only small descriptive early migration values during the first postoperative year. Larger longitudinal studies are needed to determine clinically relevant migration thresholds and the value of this method for detecting implant loosening.

## Data Availability

The data that support the findings of this study are available from Region Östergötland, Sweden, upon reasonable request. Contact the corresponding author for data requests. Restrictions may apply due to licensing and/or regulatory reasons.

## Abbreviations

ANOVA: Analysis of variance
CTMA: Computer tomography micromotion analysis
CT-RSA: Computed tomography radiostereometric analysis
EID-CT: Energy-integrating detector CT
IQRs: Interquartile ranges
PCD-CT: Photon-counting detector CT
RSA: Radiostereometric analysis
TWA: Total wrist arthroplasty
